# 

**Published:** 1927

**Authors:** 


### The Medical Library of the University of Bristol,

WITH WHICH IS INCORPORATED

**The Library of the Bristol Medico-Chirurgical Society.**

---

*The following donations have been received since the publication of the List in January,* 1927.

May, 1927.

| | |
|---|---|
| American Laryngological, Rhinological and Otological Society (1) .. .. .. .. | 1 volume |
| Dr. R. Araya (2) .. .. .. .. .. | 1 ,, |
| British Journal of Ophthalmology (3).. .. | 3 volumes |
| Col. J. Paul Bush (4) .. .. .. .. | 17 ,, |
| Dr. Wm. Cotton (5) .. .. .. .. | 7 ,, |
| Dr. D. B. Delavan (6) .. .. .. .. | 1 volume |
| Professor E. Fawcett (7).. .. .. .. | 1 ,, |
| Glasgow University (8) .. .. .. .. | 1 ,, |
| Dr. J. S. Griffiths (9) .. .. .. .. | 1 ,, |
| Professor E. W. Hey Groves (10) .. .. | 6 volumes |
| E. Herbert, Esq. (11) .. .. .. .. | 1 volume |
| Dr. G. F. Laidlaw (12) .. .. .. .. | 1 ,, |
| Michell Clarke Memorial Fund (13) .. .. | 3 volumes |
| Radium Institute (14) .. .. .. .. | 1 volume |
| Rockefeller Foundation (15) .. .. .. | 1 ,, |
| Smithsonian Institution (16) .. .. .. | 1 ,, |
| Surgeon-General, United States Army (17) .. | 1 ,, |
| Dr. J. O. Symes (18) .. .. .. .. | 1 ,, |

Unbound periodicals have also been received from Professor E. W. Hey Groves and Dr. F. S. Hunter.

### THE ONE HUNDRED AND TWENTIETH LIST OF BOOKS.

The figures in round brackets refer to the figures after the names of the donors. The books to which no such figures are attached have either been bought from the Library Fund or received through the *Journal.*

**Araya, R.** .. .. .. *Etiopatogenia y tratamiento de algunas ginecopatias* (2) 1926
**Bayly, W.** .. .. .. *Venereal Disease* .. .. .. .. .. .. 3rd Ed. 1927
**Bennett, E. N.** .. .. *With Methuen's Column on an Ambulance Train* (4) 1900
**Bowlby, A.** .. .. .. *Hunterian Oration* .. .. .. .. .. .. .. (4) 1919
**Bruce, W. I.** .. .. *A System of Radiography* .. .. .. .. .. (5) 1907
**Burke, E. T.** .. .. *Treatment of Venereal Diseases* .. .. .. .. .. 1927
**Butler, T. H.** .. .. *Illustrated Guide to Slit-lamp* .. .. .. .. .. 1927
**Bythell, W. J. S., and Barclay, A. E.** *X-Ray Diagnosis and Treatment* (5) 1912
**Cameron, H. C.** .. *Reminiscences of Lister* .. .. .. .. .. .. (8) 1927
**Chapman, C. W.** .. *The Heart and its Diseases* .. .. .. .. .. .. 1927
**Civilian War Hospital, A** .. .. .. .. .. .. .. .. .. .. .. .. .. (4) 1901
**Corner, G. W.** .. .. *Anatomical Texts of the Earlier Middle Ages* .. 1927
**Cowell, E. M.** .. .. *Hernia and Hernioplasty* .. .. .. .. .. .. .. 1927
**Dally, J. F. H.** .. .. *High Blood Pressure* .. .. .. .. .. 2nd Ed. 1926
**Delavan, D. B.** .. .. *Early Days of Presbyterian Hospital in City of New York* .. .. .. .. .. .. .. .. (6) 1926
**Diagnostiche und therapeutische Irrtümer** *Chirurgie* .. .. 8th Heft (10) 1926
**Dickson, W. K. L.** .. *The Biograph in Battle* .. .. .. .. .. .. (4) 1901
**Duchange, R.** .. .. *L'Anesthêsie Tronculaire des Machoires* (10) 1926
**Duke-Elder, W. S.** .. *Nature of the Intra-Ocular Fluids* .. .. (3) 1927
**Eason, J.** .. .. .. *Exophthalmic Goitre* .. .. .. .. .. .. .. .. 1927
**Finzi, W. S.** .. .. *Radium Therapeutics* .. .. .. .. .. .. (5) 1913
**Griffiths, J. S.** .. .. *Health and Personality* .. .. .. .. .. .. (9) 1926
**Guillain, G.** .. .. *Études Neurologiques* .. .. .. 2nd Series 1925
**Hale-White, W.** .. *Materia Media, Pharmacy, etc.* .. 18th Ed. 1924
*Health of the School Child by Various Authors* .. .. .. .. .. .. .. .. 1927
**Heines, B.** .. .. .. *Versuche über Knockenregeneration* .. (10) 1926
**Irwin, W. K.** .. .. *Urinary Surgery* .. .. .. .. .. .. 2nd Ed. 1927
**Jorden, E.** .. .. .. *A Discourse of Naturall Bathes, etc.* .. .. .. 1631
**Laidlaw, G. F.** .. .. *Treatment of Hay Fever* .. .. .. (12) 1917
**Lees, D.** .. .. .. *Diagnosis and Treatment of Venereal Diseases* .. 1927
*Lister and the Lister Ward in the Royal Infirmary of Glasgow* .. .. .. 1927
*Lister Centenary at the Wellcome Historical Medical Museum* .. .. .. 1927
**Macalpine, J. B.** .. .. *Cystoscopy* .. .. .. .. .. .. .. .. .. .. .. 1927
**Macdowall, R. J. S.** .. *Clinical Physiology* .. .. .. .. .. .. .. .. 1927
**Mackintosh, D. J.** .. *Skiagraphic Atlas of Fractures* .. .. .. (5) 1899
**MacNalty, A. S.** .. *Epidemic Diseases of the Central Nervous System* 1927
**Manson-Bahr, P. H., and Alcock, A.** *Life and Work of Sir Patrick Manson* 1927
**Meakins, J. C., and Davies, H. W.** *Respiratory Function in Disease* .. .. 1925
**Means, J. H.** .. .. *Dyspnœa* .. .. .. .. .. .. .. .. .. .. .. 1924
*Medical, Dental and Pharmacy Directory of South Africa,* 1926-7 .. (11) 1927

*Medical Department of U.S. Army in the World War.* Vol. VI. .. (17) 1926
**Nightingale, F.** .. .. *Notes on Nursing* .. .. .. .. .. .. .. .. (4) —
*Nouveau traité de médécine. Tome IX.* .. .. .. .. .. .. .. (13) 1927
**Osler, W.** .. .. .. .. *Principles and Practice of Medicine* 10th Ed. (13) 1925
**Pinch, A. E. H.** .. *Superficial Radium Therapy* .. .. .. .. .. (14) 1927
**Price, F. W.** .. .. *Diseases of the Heart* .. .. .. .. .. 2nd Ed. 1927
**Purves-Stewart, J.** .. *Diagnosis of Nervous Diseases* .. 6th Ed. (13) 1924
**Quain, J.** (Ed.) .. .. *Series of Anatomical Plates.* 5 Vols. .. .. 1836–42
*Red Cross in Gloucestershire.* 1916. 1914–19 .. .. .. .. .. .. .. (4) —
**Resnick, L., and Carris, L. H.** *Eye Hazards in Industry* .. .. .. .. .. 1924
**Scheffler, H.** .. .. *Beobachtungen und Ergebnisse bei einer fünfjährigen Frakturenbehandlung* .. .. .. .. .. .. (10) 1927
**Shenton, E. W. H.** .. *Disease in Bone* .. .. .. .. .. .. .. .. (5) 1911
*Short History of Bristol Royal Infirmary* .. .. .. .. .. .. .. .. (4) 1912
**Silverman, S. L.** .. *Principles and Practice of Oral Surgery* .. .. 1927
**Simpson, W. J.** .. .. *A Treatise on Plague* .. .. .. .. .. .. .. .. 1905
**Stern, B. J.** .. .. *Should we be Vaccinated ?* .. .. .. .. .. .. 1927
**Thomson, A., and Miles, A.** *Manual of Surgery.* Vol. 3, 7th Ed. .. (10) 1926
*United States Army X-Ray Manual* .. .. .. .. .. .. .. .. .. .. (5) 1920
**Wadd, W.** .. .. .. *Observations on Strictures in the Urethra* .. (4) 1811
**Wallace, J. S.** .. .. *Variations in the Form of the Jaws* .. .. .. 1927
*Wellcome Historical Medical Museum* .. .. .. .. .. .. .. .. .. .. .. 1927
**White, W.** .. .. .. *Observations on Strictures* .. .. 2nd Ed. (4) 1815
**Witherbee, W. D., and Remer, J.** *X-Ray Dosage* .. .. .. .. .. .. (5) 1922
**Woodwark, A. S.** .. *Manual of Medicine* .. .. .. .. .. 3rd Ed. 1927
**Wright, H. C.** .. .. *Marriage and Parentage* .. .. .. .. .. (4) 1861
**Wyard, S.** .. .. .. *Handbook of Diseases of the Stomach* .. .. .. 1927

### TRANSACTIONS, REPORTS, JOURNALS, ETC.

Annual Report of the Smithsonian Institution, 1925 .. .. .. .. .. (16) 1926
*7e Congrès de la Société Internationale de Chirurgie* .. .. .. .. .. (4) 1926
General Index to Minutes of General Medical Council, 1903–26 .. .. 1927
Guy's Hospital Reports. Vol. 77. 1. .. .. .. .. .. .. .. .. .. .. .. 1927
International Medical Congress (9th) .. .. .. .. .. .. .. .. .. .. (4) —
Medical Annual, 1927 .. .. .. .. .. .. .. .. .. .. .. .. .. .. .. .. 1927
Minutes of General Medical Council. Vol. 63, 1926 .. .. .. .. .. .. 1927
Progressive Medicine. Vol. 1, 1927 .. .. .. .. .. .. .. .. .. .. .. 1927
Report of Medical Research Council for 1925–26 .. .. .. .. .. .. 1926
Statistical Report of the Health of the Navy, 1924 .. .. .. .. .. (7) 1927
Surgical Clinics of North America. VII., i. .. .. .. .. .. .. .. .. .. 1927
Transactions of American Laryngological, Rhinological and Otological Society .. .. .. .. .. .. .. .. .. .. .. .. .. .. .. .. (1) 1926
Transactions of Association of American Physicians. Vol. XLI. .. .. 1926
Transactions of American Opthalmological Society. Vol. 24, 1926 .. .. 1926
Transactions of Ophthalmological Society of United Kingdom. Vol. 46, 1926 .. .. .. .. .. .. .. .. .. .. .. .. .. .. .. .. .. .. 1927

### Local Medical Notes.

Lieut.-Col. V. B. Green-Armytage, I.M.S., has been appointed Senior Professor of Gynæcology and Obstetrics in the University of Calcutta, and has been elected Fellow of the Royal College of Physicians, London.

**Royal College of Surgeons of England.**—E. Miles Atkinson, F.R.C.S., of Bath, has been awarded the Jacksonian Prize of the Royal College of Surgeons of England for his dissertation on "The Pathology, Diagnosis, and Treatment of Abscess of the Brain." The subject for the year 1928 is "The Surgical Treatment of Pulmonary Tuberculosis."

Robert Gordon Wilbond, of Bristol, has been awarded the Begley Studentship of the Royal College of Surgeons of England, which is given to the candidate who obtains the highest number of marks in the Anatomical part of the First Professional Examination of the Conjoint Board.

EXAMINATION RESULTS.

**University of Bristol.**—Students of the University have recently passed the following examinations :—

M.B., Ch.B. (*Part I.*).—*Second Examination. Pass :* L. P. Ashton, K. P. Beaubrun, J. E. Brown, G. E. Chadd, H. L. Cleave, L. R. Jordan, J. A. Kersley. *In Organic Chemistry* (*Completing Examination*) *:* Annie M. B. Walker. *In Elementary Anatomy only :* J. L. S. Coulter, W. A. F. Taylor. *In Organic Chemistry only :* L. C. Holland, Elizabeth S. G. Owen.

M.B., Ch.B. (*Part II.*).—*Second Examination. Pass :* J. F. Coates, Eveline M. D. M. Collinson, J. J. Giraldi (with distinction in Anatomy), G. F. Langley, R. A. Lattey, R. P. Lucas (with distinction in Anatomy), J. E. Newton, H. M. Strover, S. C. Wake (with distinction in Anatomy).

B.D.S.—*Second Examination* (*Dental Anatomy only*). *Pass :* Muriel E. S. Cosh, W. Forsyth.

DIPLOMA IN DENTAL SURGERY. — *First Professional Examination. Pass: In Dental Anatomy (Completing Examination):* T. A. Satchwell, Barbara J. Shapland. *In Anatomy and Physiology only:* I. W. Williams. *In Dental Anatomy only:* K. V. M. Taylor-Milton.

L.D.S., R.C.S.—*First Professional Examination. Part I. (Dental Mechanics):* H. J. Phillips. C. Haskins. *Part III. (a) (Anatomy and Physiology):* F. G. Lilley. *Part III. (b) (Dental Anatomy):* M. E. Minton, R. A. Phillips, J. P. Price. *Final:* J. F. V. Sellin, R. G. J. Tovey.

L.S.A.—*Medicine:* S. K. Rigg. *Anatomy and Physiology:* K. I. Nicholls.

